# Prevalence, clinical characteristics and pattern of distribution of seasonal corona virus associated acute respiratory tract infections among adults and children in the Central Province of Sri Lanka from January 2020 to October 2022

**DOI:** 10.1186/s12879-025-12458-1

**Published:** 2025-12-31

**Authors:** Shiyamalee Arunasalam, Rohitha Muthugala, Faseeha Noordeen

**Affiliations:** 1 Diagnostic and Research Virology Laboratory, Department of Microbiology, Faculty of Medicine, University of Peradeniya, Peradeniya, 20400 Sri Lanka; 2 Virology Laboratory, National Hospital Kandy, Kandy, 20000 Sri Lanka

**Keywords:** Seasonal corona viruses, Prevalence, Distribution, Clinical characteristics, Sri Lanka

## Abstract

**Background:**

Human coronaviruses (hCoVs) are frequently detected in nasopharyngeal samples from patients with acute respiratory tract infections (ARTIs). Interest in hCoVs increased following the severe acute respiratory syndrome coronavirus (SARS-CoV) outbreak in 2002. However, epidemiological data on seasonal coronaviruses (sCoVs) remain incomplete, particularly in settings where these viruses are not routinely tested in standard respiratory diagnostic panels. Limited surveillance of sCoVs may hinder early detection of emerging CoVs and pandemic preparedness.

**Methods:**

In the current study, a total of 1062 respiratory samples from patients with ARTIs were tested to detect 22 respiratory pathogens including sCoVs using a real time reverse transcription multiplex polymerase chain reaction (real time RT-multiplex PCR) from January 2020 to October 2022.

**Results:**

Respiratory pathogens were identified in 51.03% patients with the detection rate of 7% for sCoVs. Of the sCoV positive patients, 36 were hCoV-NL63/HKU1 infections; 29 were hCoV-229E infections and 9 were hCoV-OC43 infections. Fever, cough and sore throat were the most common symptoms detected in all three sCoV infections. hCoV-229E and hCoV-NL63/HKU1 were not detected in 2020. A major peak of hCoV-229E infection was noted in April 2021 with detection from January 2021 to July 2022. Major peaks of hCoV-NL63/HKU1 infections were noted in April 2021 and 2022. The least prevalent sCoV in the study was hCoV-OC43, which was detected in January to March in 2020 and the virus was not detected in 2021 and two hCoV-OC43 infections were detected in 2022.

**Conclusion:**

Based on the present study findings, prevalence of sCoV infections among patients with ARTI was 6.96% with hCoV-NL63/HKU1 predominance. sCoVs were detected year-round, with peak incidence noted in January and February of 2021/2022. sCoVs distribution fluctuated along with SARS-CoV-2 infections during the pandemic. Implementing a national sCoV surveillance system could enhance early detection and monitoring of sCoVs, aiding the tracking of emerging hCoVs.

**Supplementary Information:**

The online version contains supplementary material available at 10.1186/s12879-025-12458-1.

## Background

Coronaviruses (CoVs) are members of the family *Coronaviridae* and subfamily *Coronavirinae* and the order *Nidovirales* [1]. CoVs cause respiratory and intestinal infections in animals and humans [2]. Based on the genomic structures and the phylogenetic relationship, the subfamily *Coronavirinae* has four genera including alpha (α), beta (β), gamma (γ) and delta (δ) CoVs. α-CoV and β-CoV infect only mammals while γ-CoV and δ-CoV infect birds and mammals [3]. CoVs are the largest known RNA viruses, with a single-stranded, positive-sense RNA genome ranging from 26 to 32 kb [3].

Human coronavirus (hCoV) belongs to α and β genera of the subfamily *Coronavirinae*. hCoV was initially discovered in 1960s as the agent of common cold and detected in nasopharyngeal samples from patients with respiratory tract infections [4]. The 2002 severe acute respiratory syndrome coronavirus (SARS-CoV) outbreak heightened research interest in CoVs, leading to the identification of new subtypes such as hCoV-NL63 and hCoV-HKU1 [5]. So far, seven types of hCoVs have been identified: hCoV-229E, hCoV-NL63, hCoV-OC43, hCoV-HKU1, SARS-CoV, Middle East respiratory syndrome CoV (MERS-CoV) and SARS-CoV-2. Apart from SARS-CoV, MERS-CoV and SARS- CoV-2, other hCoVs cause seasonal outbreaks and are called sCoVs [6].

Overall global prevalence of sCoVs in respiratory tract infections is between 0.5 and 18.4% [6]. sCoV infections are associated with acute respiratory tract infection/illness (ARTI), pneumonia and croup, eventually leading to hospitalization [5]. hCoV-229E and hCoV-OC43 are transmitted during winter season in temperate countries and cause common cold like illness in infected individuals [2]. hCoV-NL63 infections peak during summer, spring and winter and infected individuals present with coryza, conjunctivitis, fever and bronchitis [7]. hCoV-HKU1 is also associated with acute exacerbation of asthma in some individuals [8]. Although hCoVs such as hCoV-229E, hCoV-OC43, hCoV-NL63 and hCoV-HKU1 cause mild infections across a wide age range, severe disease has been reported in children, elderly and immunocompromised individuals leading to hospitalization [9].

Since the emergence of SARS-CoV-2, the pattern of common respiratory infections started to change and an atypical seasonality was noted for sCoV infections [10, 11]. This change in the pattern of common respiratory infections can be partially attributed to the morphology of the virus, size, survival time on surfaces, antibody mediated cross-protection in the host, viral interference caused by interferon-stimulated immunity and COVID-19 restrictions [10]. Moreover, SARS-CoV-2 and sCoV co-infections were also noted during the pandemic [10, 12].

There are a few long-term studies available on the prevalence, clinical characteristics and pattern of distribution of sCoV globally [13–15]. Hence, limited availability of evidence has led to an incomplete epidemiology and clinical characteristics data for sCoV infections. Using longitudinal studies, the pattern of circulation of sCoV subtypes can be identified. In this study, we examined the prevalence, clinical characteristics and distribution of hCoV-229E, hCoV-OC43 and hCoV-NL63/HKU1 in patients with ARTI across all ages for 34 months in the Central Province of Sri Lanka.

## Methods

### Study design and setting

This is a descriptive study conducted at the Virology Laboratory of the National Hospital, Kandy (NHK), Sri Lanka from January 2020 to October 2022 during the COVID-19 pandemic. All respiratory samples (nasopharyngeal / oropharyngeal swab samples) received from patients with ARTI symptoms (*n* = 1062) including fever ≥ 38 °C, cough, cold, sore throat and shortness of breath (SOB) within first 7 days of the illness were selected for the study. The samples were stored at -80 °C freezer and analyzed on a weekly basis. Of the 1062 samples, 1021 were prospectively collected from January 2021 to October 2022. The rest (*n* = 41) were retrospectively analyzed from the laboratory records. These 41 samples were collected from January to December 2020. In 2020, only 41 samples were received for common respiratory viral testing at the Virology Laboratory, NHK, and all these samples were included in the study.

Ethical approval for the study was obtained from the Ethical Review Committee of the Faculty of Medicine, University of Peradeniya, Sri Lanka (Permit No: 2021/EC/21) and informed consent was obtained from all subjects and/or their legal guardian(s) prior to sample collection. All methods including data and sample collection for the study were carried out in accordance with relevant guidelines and regulations. Permission for conducting the research and data collection was also obtained from the Director, NHK, Sri Lanka.

### Collection and processing of samples

Nucleic acid extraction from the samples was done using QIA Symphony nucleic acid extraction system (Qiagen, Hilden, Germany). The nucleic acid extracts were tested for SARS-CoV-2 by real time RT-PCR (Bioneer, Catalog No: nSCV-2112, South Korea or Altona, Real Star, Cat No: 015,821, Germany) and other common respiratory pathogens [respiratory syncytial virus-A, B (RSV-A, B), influenza-A, B, H1N1 (inf-A, B, HINI pdm 09), human parainfluenza virus- 1 to 4 (hPIV-1 to 4), hCoV (hCoV OC43,NL63/HKU1,hCoV 229E), Rhinovirus/Enterovirus (Rh/EnV), human adenovirus (hAdV), human metapneumovirus (hMPV), human bocavirus type-1 (hBoV-1) and four atypical bacteria including *Mycoplasma pneumonia* (*M. pneumoniae*), *Chlamydophila pneumoniae* (*C. pneumoniae*), *Legionella pneumophila* (*L. pneumophila*), *Bordetella* species (*Bordetella spp*)] by a commercial real time PCR assay (Respifinder2SMART, Catalog No: PF2600-2 S, Netherlands) as per manufacturer’s instructions. Samples collected from January to December 2020 were tested only for common respiratory pathogens using commercial real time reverse transcriptase multiplex PCR assay (Respifinder 2SMART, Catalog No: PF2600-2 S, Netherlands).

### Principles of respifinder^®^ 2SMART assay

The RespiFinder^®^ 2SMART assay is based on the SmartFinder^®^ technology, which allows a highly complex analysis of up to 13 targets in a single PCR reaction. The assay contains 23 different 2SMART primer sets targeting pathogen specific genes combined with 15 fluorescent labelled SMART probes, which detects pathogens and controls (Additional file). This starts with a pre-amplification reaction, which combines a RT step with a PCR step to amplify the target cDNA (Tables 1 and 2). Subsequently, a part of the pre-amplification reaction mixture is transferred to two PCR tubes and two separate SmartFinder^®^ reactions are performed (Tables 3 and 4). The final pathogen detection is performed using a melting curve analysis. RespiFinder^®^ 2SMART uses a Rotor-Gene^®^ instrument (Corbett Life Science, Australia) for the nucleic acid detection of pathogens. Three different channels are used for the acquisition of different fluorescent signals (ROX, Cy5 and FAM).

**Table 1:** Constituents of pre-amplification reaction mix of the RespiFinder^®^2SMART

Components	Volume (µl)
Pre-amplification Master mix	6.25
Pre-amplification primer mix	8.75
Template	10

**Table 2:** Thermal conditions used for pre-amplification in the RespiFinder^®^2SMART

Tm (^o^C)	Time (minute: second)	No. of cycle
50	10:00	1
95	2:00	1
94	0:10	40
55	0:30	
72	0:35	
20	Hold	

**Table 3:** Constituents of generic-amplification reaction mix of the RespiFinder^®^ 2SMART assay

2SMART mix	Components	Volume (µl)
1	2SMART buffer 1	19
	Taq polymerase	1
	Pre-amplification reaction product	5
2	2SMART buffer 2	19
	Taq polymerase	1
	Pre-amplification reaction product	5

**Table 4:** Thermal conditions for 2SMART Step-2 reaction of the RespiFinder^®^ 2SMART assay

Tm (^o^C)	Time (minute: second)	No. of cycle
95	02:00	1
94	00:15	10
55	00:15	
72	00:15	
94	00:15	23
50	00:15	
72	00:15	
95	02:00	1

## Results

**Fig. 1:**
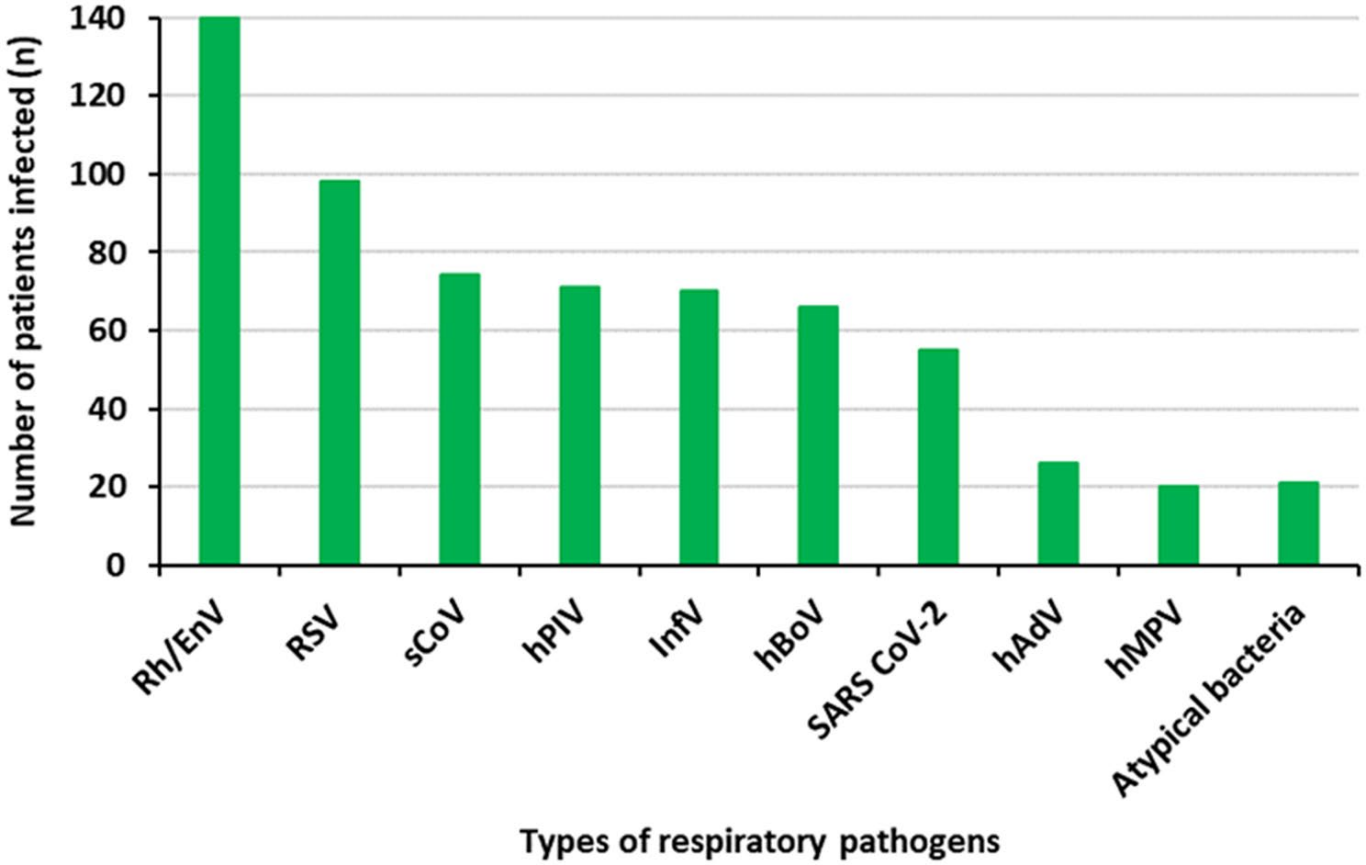
The overall prevalence of respiratory pathogens in the study sample. sCoVs were the third predominant respiratory viruses detected in the study population, 74 out of 1062 patients

Of the 1062 patients’ samples tested, 51.03% (542/1062) were positive for atleast one respiratory pathogen. Of the respiratory pathogen positive patients, 83.4% (452/542) had single infections and 16.6% (90/542) had co-infections. Of the 51.03% (542/1062) patients positive for any of the respiratory infections, 25.83% (140/542) had Rh/EnV; 18.08% (98/542) had RSV-A/B and 10.14% (55/542) had SARS-CoV-2. In addition, 13.09% (71/542) had hPIV-1-4; 12.91% (70/542) had inf-A/B; 12.17% (66/542) had hBoV-1; 4.79% (26/542) had hAdV and 3.6% (20/542) had hMPV (Additional file). Moreover, 3.87% (21/542) had atypical bacteria (*Bordetella spp* / *L. pneumophila* / *M. pneumoniae* / *C. pneumoniae*). sCoVs (hCoV-229E/ NL63/ HKU1/ OC43) were detected in 13.65% (74/542) patients (Fig. 1).

**Fig. 2:**
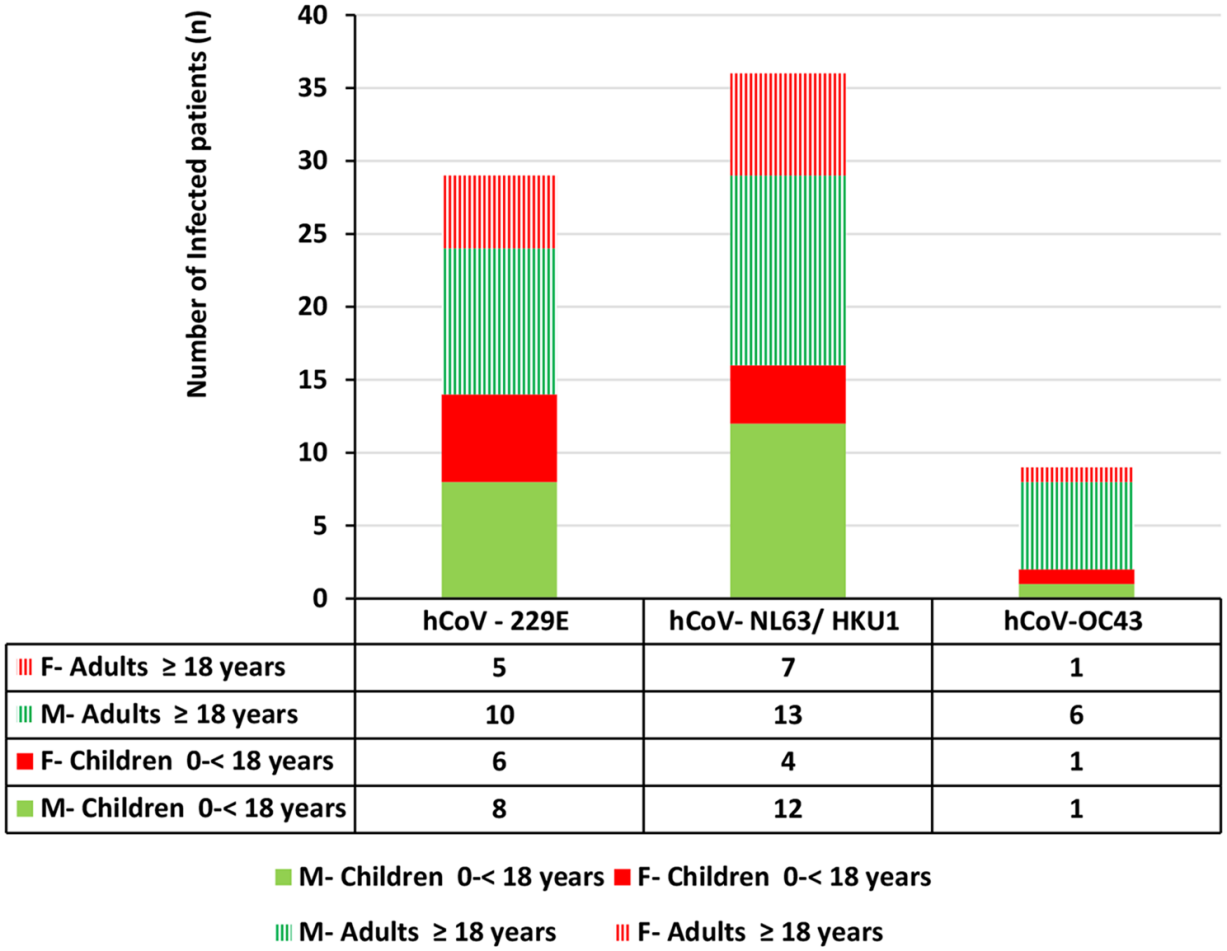
Distribution of sCoVs with relevance to age and sex of the study participants. All three sCoVs were detected commonly in adults compared to children with male predominance

The median age of sCoV-positive patients was 17 years, (IQR = 46) and all three sCoVs were commonly detected in adults compared to children with male predominance (Fishers exact test, *p* = 0.709) (Fig. 2). Of the sCoV positive patients, 48.64% (36/74) had hCoV-NL63/HKU1 infection, 39.18% (29/74) had hCoV-229E infection and 12.16% (9/74) had hCoV-OC43 infection. 68.91% patients (51/74) had sCoV single infection and 31.08% (23/74) had co-infections with any of the other respiratory pathogens tested. Rh/EnV and hAdV are the predominant viruses co-infecting with sCoVs. Moreover, 4 patients were co-infected with SARS- CoV-2 (Table 5).

Fever was observed in 86.5% (64/74) of patients, cough in 75.7% (56/74), and sore throat in 48.6% (36/74), making them the most prevalent symptoms across all three sCoV infections. Shortness of breath (SOB) (30%) and diarrhoea (11%) were the least frequently reported symptoms. Eight patients had lower respiratory tract infection (LRTI) (11%, 8/74) and intensive care was needed for four patients (5.4%, 4/74) (Table 6).

**Fig. 3:**
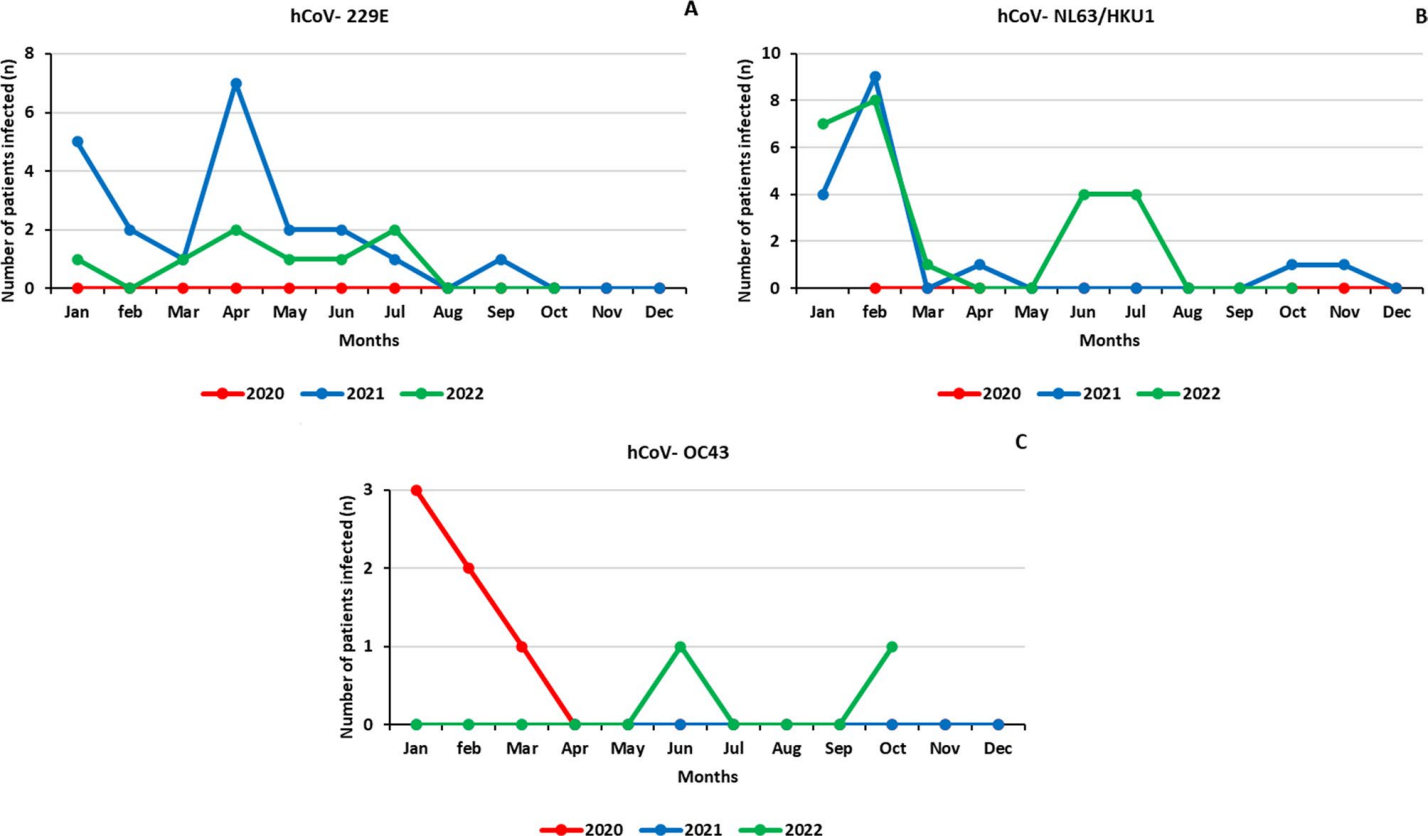
Distribution of sCoVs in the study sample from January 2020 to October 2022 (**A**: Distribution of hCoV-229E; **B**: Distribution of hCoV-NL63/HKU1; **C**: Distribution of hCoV-OC43). No hCoV-229E infection was detected in 2020 and a major peak was noted in April 2021. hCoV-229E was prevalent from January 2021 to July 2022 and hCoV-NL63/HKU was not detected in 2020. Major peaks of hCoV-NL63/HKU were noted in April 2021 and 2022. The least prevalent sCoV was hCoV-OC43, which was detected from January to March in 2020 and not detected in 2021. There were only two hCoV- OC43 infections detected in 2022

Monthly distribution of sCoVs is shown in Fig. 3. hCoV-229E infection was absent in 2020 and the major peak for hCoV-229E infection was observed in April 2021. hCoV-229E was prevalent from January 2021 to July 2022. Compared to 2021, prevalence of hCoV-229E infection was less in 2022. hCoV-NL63/HKU1 infection was not detected in 2020. Major peaks of hCoV-NL63/HKU1were noted in April 2021 and 2022. Prevalence of hCoV-NL63/HKU1 infection was higher in 2022 than that noted in 2021. The least prevalent sCoV was hCoV-OC43 and it was detected from January to March in 2020 and this virus was not detected in 2021 and only two hCoV-OC43 infections were detected in 2022.

**Fig. 4:**
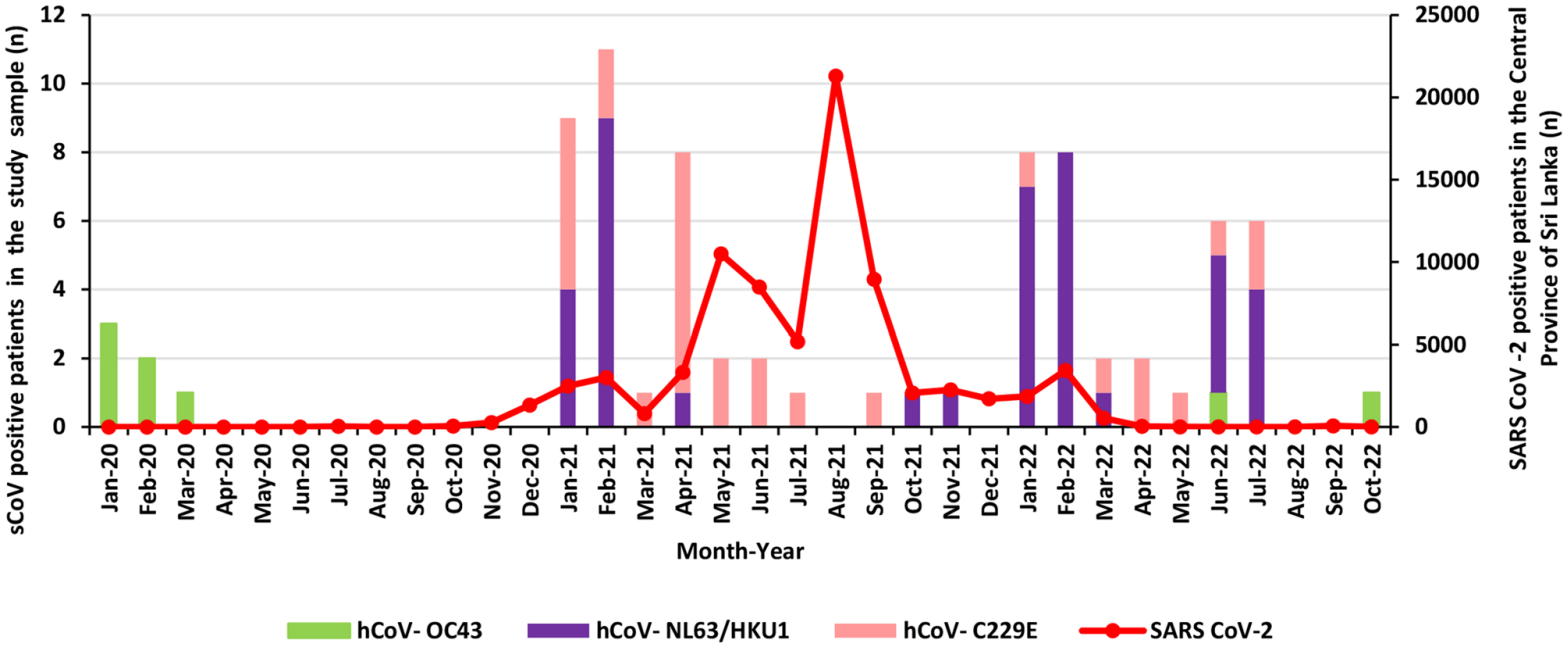
Distribution of sCoVs along with SARS-CoV-2 infections from January 2020 to October 2022 in the Central Province of Sri Lanka. hCoV-OC43 was prevalent during the early and declining stages of theCOVID-19 pandemic. From April 2020 to June 2022, no hCoV-OC43 infection was detected. hCoV-C229E was the only sCoV subtype detected from May to September in 2021 and no sCoV infections was detected in August 2021. In most of the months in 2021 and 2022, both hCoV-229E and hCoV-NL63 infections were detected

Figure 4 shows the distribution of sCoVs along with COVID-19 cases in the Central Province of Sri Lanka. hCoV-OC43 was detected before the COVID-19 waves (1st wave 27th of January to 3rd of October 2020; 2nd wave 4th of October 2020 to 14th of April 2021; 3rd wave 15th of April 2021 to 30th of September 2022) and during the declining stage of the pandemic in 2022. hCoV-OC43 was not detected between April 2020 and June 2022. hCoV-229E was the only sCoV subtype detected from May to September in 2021 when the major COVID-19 peak was noted. However, none of the sCoVs was detected in August 2021; hCoV-229E and hCoV-NL63 co-circulated in several months in 2021 and 2022.

**Table 5:** sCoV co-infections (*n* = 23) detected among sCoV positive patients (*n* = 74)

Co-infections	Viral types	Subtypes	Number	Total
Dual infection	sCoVs + SARSCoV-2	hCoV-229E + SARSCoV-2	3	5
		hCoV-NL63/HKU1 + SARSCoV-2	2	
	sCoVs + hBoV	hCoV-229E + hBoV-1	1	1
	sCoVs + InfV	hCoV-OC43 + Inf-A	1	1
	sCoV + RSV	hCoV-229E + RSV-B	1	2
		hCoV-NL63/HKU1 + RSV-A, -B	1	
	sCoVs + Rh/EnV	hCoV-NL63/HKU1 + Rn/EnV	5	6
		hCoV-229E + Rh/EnV	1	
	sCoVs + hPIV	hCoV-229E + PIV-4	1	1
	sCoVs + hAdV	hCoV-229E + hAdV	4	6
		hCoV-NL63/HKU1 + hAdV	2	
	sCoVs + hMPV	hCoV-OC43 + hMPV	1	1
Triple infection	sCoVs + hBoV-1 + atypical bacteria	hCoV-229E + hBoV-1 + *M. pneumoniae*	1	1
**Total sCoV co-infections**				**23**

EnV- Enterovirus, hAdV- Human adenovirus, hBoV-1- Human bocavirus-1, hCoV- Human coronavirus, hMPV- Human metapneumovirus, hPIV- Human parainfluenza virus, InfV- Influenza virus, *L. pneumophila* - *Legionella pneumophila*, *M. pneumoniae* - *Mycoplasma pneumoniae*, RhV- Rhinovirus, RSV- Respiratory syncytial virus, SARSCoV-2- Severe acute respiratory syndrome corona virus-2, sCoV- Seasonal coronavirus

**Table 6:** Demographic and clinical characteristics of sCoV infections in the study sample

Characteristics	hCoV-229E *n* = 29 (2.8%)	hCoV-NL63/ HKU1 *n* = 36 (3.5%)	hCoV-OC43 *n* = 9 (0.84%)	Total *n* = 74 (6.96%)
**Demographic characteristics**				
Gender (M: F)	17:12	25:11	7:2	25:49
Adult: Children	14:15	21:15	7:2	42:32
**Clinical characteristics**				
Fever	26 (89.65%)	30 (83.33%)	8 (88.88%)	64 (86.48%)
Cough	19 (65.51%)	29 (80.55%)	8 (88.88%)	56 (75.67%)
Sore throat	16 (55.17%)	18 (50%)	1 (11.11%)	35 (47.29%)
Shortness of breath	12 (41.37%)	9 (25%)	1 (11.11%)	29 (39.18%)
Diarrhea	4 (13.79%)	4 (11.11%)	0 (0%)	8 (6.75%)
Co-morbidities	3 (10.34%)	2 (5.55%)	0 (0%)	5 (6.75%)
Need for intensive care management	2 (6.89%)	1 (2.77%)	1 (11.11%)	4 (5.40%)

Numerical data are given as mean, categorical variables are expressed as percentages. hCoV - Human coronavirus

## Discussion

The present study findings describe the prevalence, demographic patterns, and clinical presentation of sCoV infections in children and adults from January 2020 to October 2022. This is the first large scale study with sCoV subtypes (hCoV- 229E, NL63/HKU1 and OC43) circulated in Sri Lanka during the study period.

The overall prevalence of sCoV infections in the study sample was 6.96%. This is considerably higher than previously reported rates in Sri Lanka, particularly before the COVID-19 pandemic in a few small scale studies [16, 17]. The study by Saphiro et al. reported 1.7% prevalence for sCoV infections among adults and children in the Southern part of Sri Lanka from March 2013 to January 2015 [16]. Another study done by Jayaweera et al. in the North Central and Central provinces of Sri Lanka from March 2013 to August 2014 did not find sCoV infections in their study populations [17]. Our study found a significantly higher detection rate of sCoVs in alignment with the findings of Sathgurupathi et al. (2021), compared to pre-COVID-19 levels. Sathgurupathi et al. reported 40% prevalence of sCoV infections among 384 patients with ARTI using samples collected from January to March 2021 in the North Central part of Sri Lanka [18]. Increase in the prevalence of sCoV infections may be due to heightened surveillance, improved diagnostics, or changes in viral circulation dynamics.

Among the sCoV-positive patients, males exhibited a higher prevalence, a trend consistent with previous reports on other respiratory viral infections [19–21]. In contrast to findings of the previous studies, sCoV infections were more prevalent among adults than children in our study [22–24].

It has to be noted that there is a substantial variation in the prevalence of sCoV subtypes in different epidemiological studies that included patients with ARTI [10, 14, 25, 26]. The predominant sCoV detected in this study was hCoV-NL63/HKU1 followed by hCoV-229E and hCoV-OC43. However, hCoV-OC43 was the predominant sCoV detected in a previous study conducted in the Southern part of Sri Lanka and other parts of the world including the United Kingdom and Hong Kong before the COVID-19 pandemic [15, 16, 27]. A study done by Heimdal et al., before the COVID-19 pandemic among hospitalized Norwegian children with ARTI for nine years, also reveals that hCoV-OC43 was the most commonly detected subtype [28]. However, based on the observation during the pandemic, the prevalence rate of hCoV-OC43 has been reduced and this is supported by another small scale study done in Sri Lanka as well [18].

The varying prevalence of sCoVs may be attributed to several factors including the interference from the widely circulated SARS-CoV-2, non-pharmaceutical interventions implemented to control the spread of SARS CoV-2 and non-specific interference caused by interferon. Moreover, the number of samples tested during 2020 is less compared to that in 2021 and 2022 and this might be a reason for not detecting sCoVs like hCoV-229E and hCoV-NL63/HKU1 in 2020. Viral interference is one of the reasons for the shift in the prevalence between hCoV-OC43 and hCoV-NL63/HKU1. hCoV-NL63 and hCoV-HKU1 are less genetically related to SARS-CoV-2 than hCoV-OC43, possibly allowing them to co-circulate. Moreover, immune response induced by SARS-CoV-2 infection or vaccination derived temporary cross-immunity might have suppressed hCoV-OC43 than hCoV-NL63 or hCoV-HKU1.

Of the seven hCoVs, sCoVs cause symptoms similar to epidemic CoVs but with less disease severity [26, 29, 30]. However, there is some evidence to suggest that co-infection with endemic or epidemic hCoVs, sCoVs can cause severe disease [31, 32]. In our study four patients were co-infected with SARS-CoV-2 including three children and one adult. Of these, the 50-year-old adult had severe respiratory symptoms and required mechanical ventilation. Of the four SARS-CoV-2 co-infected patients, two had hCoV-229E and SARS-CoV-2 co-infections and two had hCoV-NL63/HKU1 and SARS-CoV-2 co-infections. No hCoV-OC43 and SARS-CoV-2 co-infections were detected in our study, however, studies conducted in other parts of the world document hCoV-OC43 and SARS- CoV-2 co-infections [31, 33]. The absence of hCoV-OC43 and SARS- CoV-2 co-infections in our study population might be due to very less number of hCoV-OC43 infections noted in our study during the study period from 2020 to 2022.

hCoVs are widespread globally and the pattern of distribution varies according to the region and seasonal factors [6, 34, 35]. However, sCoVs are usually detected throughout the year in tropical countries. In our study sCoVs were detected year-round with major peaks in January - February in 2021 and 2022. As our study period was within the COVID-19 pandemic, other than the seasonal factors, interference from the SARS-CoV-2, non-pharmaceutical interventions implemented to control the spread of SARS CoV-2 and non-specific interference caused by interferon may also have influenced the circulation of sCoVs [23]. In Sri Lanka, the first COVID-19 patient was identified in December 2019 and three COVID-19 waves (1st wave 27th of January to 3rd of October 2020; 2nd wave 4th of October 2020 to 14th of April 2021; 3rd wave 15th of April 2021 to 30th of September 2022) were reported by the Epidemiology Unit of Sri Lanka. During the first wave, a maximum of less than a thousand cases were reported per week and this was considerably lower than the cases reported subsequently in the next two waves. The detection rate of sCoV was less in the first wave compared to the next two years of our study (2021/2022), which mainly falls within the second and third waves. The prevalence of sCoV was higher in January and February in 2021 and this falls within the COVID-19 s wave in which increased number of COVID-19 cases were reported weekly [36].

Moreover, the prevalence of hCoV-OC43 was very low during the months when SARS-CoV-2 peak was noted. hCoV-229E and hCoV-NL63/HKU1 co-circulated during the second and third COVID-19 waves. However, hCoV-229E was the only sCoV circulated from May to September in 2021 when the most prominent SARS-CoV-2 pandemic peak was noted. No sCoVs were detected in August 2021 when the maximum number of SARS CoV-2 cases were recorded [36].

SARS-CoV-2 and sCoVs share more than 30% similarity in their genetic code within the S2 subunit and this may result in overlapping immune epitopes [37]. Studies suggest that either by vaccination or natural infection based protective immunity against the SARS-CoV-2 might have increased cross protection against sCoVs [38, 39]. However, there is a disagreement among researchers as some argue that the antibodies may increase but not effectively prevent sCoVs infections or hospitalizations [40]. Frequent recombination and declining natural immunity against hCoV suggests that any cross-protection from infection or vaccination may not offer sufficient defense against future infections [36, 41, 42]. The current study findings reveal that the co-circulation of sCoVs and SARS-CoV-2 is possible and the pattern of sCoVs subtypes suggests the effects of viral interference or cross-protection among CoVs. Additional studies are needed to comprehensively understand how SARS-CoV-2 influences the circulation of sCoVs and the complex immune interactions between these viruses [10]. It is important to note that all hCoVs use different receptors in host cells for entry and sCoVs express the spike glycoprotein, which extends from the surface of the virus. There is a significant similarity between hCoV-229E and hCoV-NL63 spike glycoproteins and this is supported by our finding that hCoV-229E and hCoV- NL63/HKU1 co-circulated in most of the months [6]. Future directions to combat the limitations of the current study include, establishing multi-center surveillance studies across diverse climates including both symptomatic and asymptomatic individuals, employing specific molecular assays, to separately identify the prevalence of hCoV-NL63 and hCoV-HKU1 infections.

## Conclusion

The present study reports a 6.96% prevalence of sCoV infections in patients with ARTI. Of the respiratory pathogen–positive patients, sCoVs (hCoV-229E, NL63, HKU1, and OC43) were detected in 13.65% (74/542). The most frequently identified sCoV subtype was hCoV-NL63/HKU1. Fever, cough, and sore throat were the most commonly reported symptoms across all three sCoV infections. sCoVs were detected throughout the year, with increased detections in January and February of 2021 and 2022. Variation in the distribution of sCoVs and their subtypes was noted during the COVID-19 pandemic and this may reflect changes in virus circulation patterns during the pandemic period. Establishing a national sCoV surveillance system could support more consistent monitoring of sCoV activity and contribute to a better understanding of circulating hCoVs.

## Supplementary Information

Below is the link to the electronic supplementary material.

Supplementary Material 1

## Data Availability

No datasets were generated or analysed during the current study.

## Declarations

### Limitations

As the present study tested samples received from the Virology Laboratory of the NHK only, these findings may not fully represent the respiratory virus circulation patterns across the Central Province of Sri Lanka. Since the study included patients with ARTI symptoms within the first 7 days, infections presenting later, with atypical symptoms, or with prolonged duration may have been missed. Moreover, the present study included samples from symptomatic patients excluding individuals with mild or asymptomatic infections who did not seek medical care and this again will underrepresent the respiratory virus circulation patterns across the Central Province of Sri Lanka. This study did not do patient follow-up, hence, correlations between viral pathogens and disease severity, duration of hospitalization, or clinical complications could not be assessed.

## Abbreviations

ARTI: Acute respiratory tract infection
Bordetella spp: Bordetella species
C. pneumoniae: Chlamydophila pneumoniae
EnV: Enterovirus
hAdV: Human adenovirus
hBoV-1: Human bocavirus-1
hCoV: Human coronavirus
hMPV: Human metapneumovirus
hPIV: Human parainfluenza virus
ICU: Intensive Care Unit
InfV: Influenza virus
L. pneumophila: Legionella pneumophila
LRTI: Lower respiratory tract infection
MERS-CoV: Middle East respiratory syndrome-CoV
M. pneumoniae: Mycoplasma pneumoniae
RhV: Rhinovirus
RSV: Respiratory syncytial virus
SARS-CoV: Severe acute respiratory syndrome-CoV
sCoV: Seasonal coronaviruses
