# Supplementary material for: Prevalence, clinical characteristics and pattern of distribution of seasonal corona virus associated acute respiratory tract infections among adults and children in the Central Province of Sri Lanka from January 2020 to October 2022
Source: BMC Infect Dis. 2025 Dec 31;26:226. doi: 10.1186/s12879-025-12458-1 (PMC12866523; doi:10.1186/s12879-025-12458-1)
Supplement: Supplementary file 1 — Supplementary Material 1 [file 12879_2025_12458_MOESM1_ESM.pdf]

Additional file

Table 7. Target genes used for the primer design of the RespiFinder® 2SMART assay.

**Target**	**Gene**
Flu A	Matrix protein (M1) gene
Flu B	M1 gene
Flu A H1N1 pdm09	Neuraminidase gene
hMPV	Nucleocapsid protein (NP) gene
hAdV	Hexon (H) gene
hPIV-1	Haemagglutinin-neuraminidase (HN) gene
hPIV-2	HN gene
hPIV-3	HN gene
hPIV-4	Major NP gene
RhV/EnV	5' untranslated region polyprotein (PP) gene
RSV-A	Nonstructural protein gene
RSV-B	Nucleoprotein gene
hCoV-NL63	NP gene
hCoV-OC43	NP gene
hCoV-229E	NP gene
hCoV-HKU1	Nucleocapsid phosphoprotein (N) gene
hBoV	NP-1 gene
B. pertussis	Insertion sequence 481
L. pneumophila	Macrophage infectivity potentiator (MIP) gene
M. pneumoniae	Cytadhesin protein (P1) gene
C. pneumoniae	Major outer membrane protein gene
Amplification control 1	Unique artificial sequence (UAS)
Amplification control 2	UAS

Table 8. Targets and corresponding temperature values of smart probes used in the RespiFinder® 2SMART assay.

**2SAMRT reaction**	**Label**	**Smart probe**	**Pathogen**	**Average Tm (°C)**	**Tm range ARTIs)**
1	ROX	Probe 1	RSV-A	55	53.5-56.5
		Probe 2	hAdV	59.5	58-61
		Probe 3	hMPV	64	62.5-65.5
		Probe 4	RSV-B	68	66.5-69.5
		Probe 5	Inf-A	74	72.5-75.5
		Probe 6	Inf-B	78	76.5-79.5
	Cy5	Probe 1	*L. pneumophila*	51.5	49.5-53.5
		Probe 2	*B. pertussis*	56.5	55-58
		Probe 3	Rh/EnV	62	60.5-63.5
		Probe 4	*C. pneumoniae*	68	66.5-69.5
		Probe 5	*M. pneumoniae*	72	70.5-73.5
	FAM	Probe 1	Amplification control 1	63	61-65
2	ROX	Probe 1	hPIV-1	55	53.5-56.5
		Probe 2	hPIV-2	59.5	58-61
		Probe 3	hPIV-3	64	62.5-65.5
		Probe 4	hPIV-4	68	66.5-69.5
		Probe 5	hBoV-1	74	72.5-75.5
	Cy5	Probe 1	hCoV-OC43	51.5	49.5-53.5
		Probe 2	hCoV-NL63/HKU1	54	52.5-55.5
		Probe 3	hCoV-229E	61	58.5-63.5
		Probe 6	Inf-A H1N1 pdm09	77.5	76-79
	FAM	Probe 1	Amplification control 2	57.5	55.5-59.5
